# Sarcoid-Like Reactive Lymphadenopathy in Metastatic Synovial Sarcoma

**DOI:** 10.7759/cureus.35648

**Published:** 2023-03-01

**Authors:** Gundip S Dhillon, Yema Jalal, Vishisht Mehta

**Affiliations:** 1 Internal Medicine, Mountainview Hospital, Las Vegas, USA; 2 Interventional Pulmonology, Mountainview Hospital, Las Vegas, USA

**Keywords:** lymph nodes pathology, noncaseating granuloma, sarcoma soft tissue, synovial sarcoma, sarcoid like reaction

## Abstract

A 56-year-old male underwent treatment for sarcoma with metastases to the lungs. Follow-up imaging revealed multiple pulmonary nodules and masses with a favorable response on positron emission tomography (PET) scanning showing enlarging mediastinal lymph nodes concerning for progression of the disease. To evaluate the lymphadenopathy, the patient underwent bronchoscopy with endobronchial ultrasound and transbronchial needle aspiration. The lymph nodes were negative for cytology but showed granulomatous inflammation. Granulomatous inflammation is a rare occurrence in patients with concurrent metastatic lesions and is exceedingly rare in cancers that have not originated in the thorax. This case report highlights the clinical significance of sarcoid-like reactions in the mediastinal lymph nodes and the need for further investigation.

## Introduction

A sarcoid-like reaction is hypothesized to be a T-cell-mediated immunologic response to malignant tumor markers causing a granuloma to form in the lymph nodes. These granulomatous reactions are often due to both infectious and non-infectious etiologies [1]. When granulomas are related to malignant tumors, it is considered a “sarcoid-like reaction” and the infectious process should be ruled out. Non-infectious etiologies of sarcoid-like reactions are mostly related to Hodgkin's lymphomas, but they can be seen in carcinomas, such as non-small cell lung cancer (NSCLC). They are, however, extremely rare in sarcomas [1]. Often lymphadenopathy from sarcoid-like reactions is very difficult to differentiate between malignancies, even on high-resolution imaging. Clinical investigation can aid in differentiating between the two, such as a thorough examination of the signs or symptoms of serious disease. The differential diagnosis for hilar lymphadenopathy includes sarcoidosis, tuberculosis, and lymphoma. A common differential for hilar lymphadenopathy includes sarcoidosis, tuberculosis, and lymphoma; although there is utility in clinical evaluation, lymph biopsy has the strongest diagnostic utility [2]. 

This case is unique because granulomatous lesions that form from sarcoid-like reactions can perplex determining and differentiating the lesion from malignant causes with metastasis from non-thoracic origins. The challenge faced is to distinguish the worsening PET-avid adenopathy from a benign cause like a sarcoid-like reaction as opposed to further progression of the disease. 

## Case presentation

A 56-year-old male with a past medical history of synovial sarcoma in the left arm was diagnosed in May of 2020. In August of that year, he was treated with radiation and then three cycles of adriamycin, ifosfamide, and mesna (AIM) chemotherapy. In May and June of 2021, he underwent a resection of the tumor twice. After the two resections, he resumed AIM chemotherapy in September of 2021 and finished another six cycles, with the last cycle in November of 2021.

**Figure 1:**
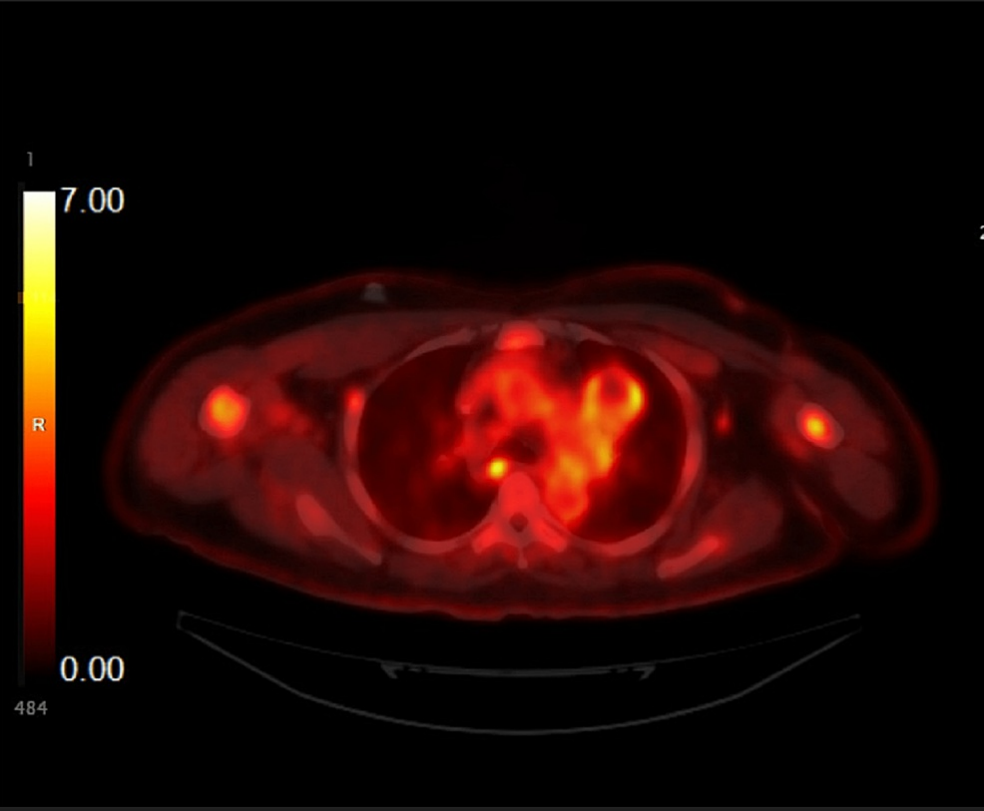
FDG-PET scan showing FDG avidity in the subcarinal LN (September 2022) FDG, fluorodeoxyglucose, PET, positron emission tomography, LN, lymph node

A computer tomography (CT) scan of the chest showed large lung masses in both hila. It was later discovered that he had metastatic sarcoma to the lungs, which was seen in pulmonary follow-up to review imaging studies. PET scans showed continued improvement of his pulmonary lesions but now also demonstrated worsening avidity in the mediastinal lymph nodes. Figure 1 was taken in September 2022. The increase in fluorodeoxyglucose (FDG) correlates with the lymphadenopathy burden, which is often confused with a cancer burden. This was concerning for the progression of the disease and malignant lymphadenopathy.

During the initial encounter after being admitted to the hospital, he denied chest pain and dyspnea and was placed on oxygen for support. He then underwent flexible bronchoscopy with endobronchial ultrasound (EBUS)-guided transbronchial needle aspiration of the PET-avid lymph nodes. The samples were sent for cytopathologic evaluation. Also, a hitherto unknown right lower lobe endobronchial lesion was also visualized, biopsied and removed entirely, and submitted for pathologic evaluation. The patient was discharged the following day and recommended close follow-up with the pulmonologist.

**Figure 2:**
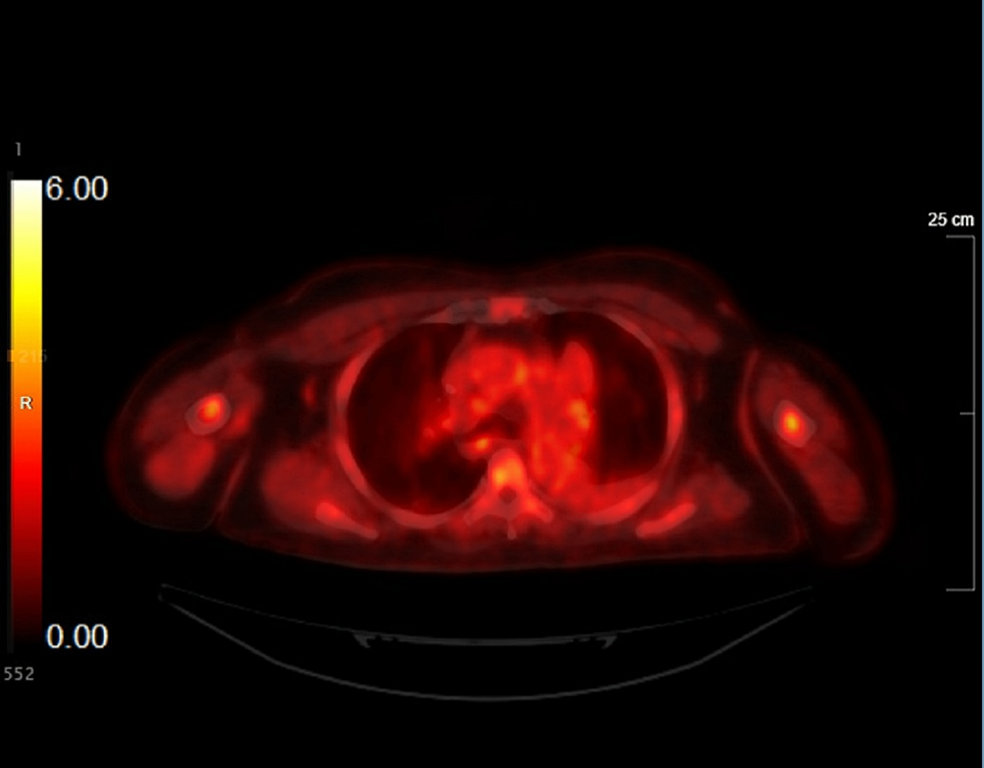
FDG-PET scan showing improving FDG avidity in the subcarinal LN (January 2023) FDG, fluorodeoxyglucose, PET, positron emission tomography, LN, lymph node

The final pathology was consistent with metastatic sarcoma; however, all submitted lymph nodes demonstrated benign findings and some showed granulomatous inflammation. Figure 2 shows decreased FDG, correlating with decreased lymphadenopathy. A following bronchoscopy done a few months later revealed tumors too small to intervene on the right lower lobe. The patient was discharged with the instructions to follow up with a repeat bronchoscopy in three to six months.

## Discussion

Synovial carcinoma is a mesenchymal tumor that is most often seen in patients below the age of 30. In order to diagnose synovial carcinoma, a biopsy of the cancer as well as radiographic tests have to be conducted. The disease is either localized or systemic, and which of the two the patient has will guide treatment. The primary treatment for localized synovial carcinoma is limb-sparing surgery along with radiation therapy. If the tumor is not amendable to resection or is metastatic, the mainstay treatment is anthracycline-based therapy [3]. 

A sarcoid-like reaction of the lymph nodes concurrently with metastatic malignancy is a rare occurrence. Sarcoidosis refers to inflammatory disease in which non-caseating granulomas develop and diagnosis is achieved by clinical presentation and exclusion of other causes of granulomas. These exclusions of other causes for sarcoid-like reactions are not limited to cancers, lymphomas, and drug-induced granulomas. Sarcoid-like reactions should be biopsied when suspected secondary to a cancer or lymphoma. Once a sarcoid-like reaction has been diagnosed, the follow-up recommendations include following up with the suspected cancer through imaging [4]. Management for treating sarcoid-like reactions secondary to cancer is similar to the treatment of sarcoidosis, which is primarily corticosteroids. Treatment should pertain to the organ affected by the sarcoid-like reaction as well [4]. A retrospective review was performed at one center evaluating all patients where granulomatous inflammation was detected on EBUS and infectious causes were excluded over a period of 32 months. In the review, they found that 7.8% (n=12) had granulomatous inflammation and a concurrent cancer diagnosis and six of these cases were positive for non-caseating granulomatous disease [5]. Ten out of the 12 cases in question had a malignancy originating at or near the thorax. Sarcoid-like reactions also differ from sarcoidosis based on their histological characteristics. Sarcoid-like reactions and sarcoidosis are both T-cell mediated; however, they differ in the size and number of multinucleated cells [6]. This was studied by comparing sarcoidosis and sarcoid-like reactions caused by lung cancer. They also showed that sarcoidosis is caused by a host reaction of foreign substances and tended to have *P. Acnes* appear more frequently on biopsy results. 

The literature describes patients who have granulomatous disease originating near the thorax or from primarily lung cancer. In our patient, the malignancy originated in the right upper extremity and was musculoskeletal in origin. Sarcoid-like reactions are also associated with immunotherapy, occurring either in the tumor of origin or from tumor-draining lymph nodes. There are also a few reports of sarcoid-like reactions secondary to sarcomas [7]. 

According to Ravaglia et al., radiolabeled glucose molecules can be picked up on PET scans, whether it is a cancer originating in the thorax or a sarcoid-like reaction from lymphadenopathy. After a positive PET scan, in order to further investigate and differentiate between the two, histologic biopsies need to be obtained [7]. Guidelines are unclear on whether this phenomenon can be beneficial, and information on survival predictability is scarce. A study was conducted comparing patients with granulomatous inflammation with cancer to those with benign mediastinal lymphadenopathy. 106 patients were reviewed, with a 90% 3-year survival for those who had granulomatous inflammation compared to 88% 3-year survival for those who had benign inflammation [8]. Overall, the results were not significantly different. But there are other studies that reported sarcoid-like reactions associated with non-small cell lung cancer were associated with better outcomes in terms of survival [9].

Although mediastinal lymphadenopathy is common in patients with malignancy and warrants biopsy in order for staging, granulomatous inflammation should be considered, and guidelines should reflect this possible diagnosis. 

## Conclusions

Sarcoid-like reactions in the context of metastatic cancer are rare, and they are rarer still when associated with extra-thoracic cancers. There are studies that have shown findings more associated with sarcoid-like reactions and early staging of cancers. It is important to recognize that along with metastatic adenopathy, sarcoid-like reactions can co-exist with malignancies when evaluating PET-avid lymph nodes. 
